# East–west contrasting changes in Southern Indian Ocean Antarctic Bottom Water salinity over three decades

**DOI:** 10.1038/s41598-022-16331-y

**Published:** 2022-07-16

**Authors:** Yeon Choi, SungHyun Nam

**Affiliations:** 1 School of Earth and Environmental Sciences, College of Natural Sciences, Seoul National University, Seoul, 08826 Republic of Korea; 2 Research Institute of Oceanography, College of Natural Sciences, Seoul National University, Seoul, 08826 Republic of Korea

**Keywords:** Physical oceanography, Physical oceanography

## Abstract

Antarctic Bottom Water (AABW) characteristics, derived from multiple water sources with various properties, are significantly affected by and contribute to climate change. However, the underlying causes of changes in AABW characteristics are not well-understood. In this study, we aimed to analyse the east–west contrasting pattern of AABW characteristics in the Southern Indian Ocean (SIO) over the last three decades. We show that AABW has become warmer and more saline in the western SIO (WSIO) but warmer and fresher in the eastern SIO (ESIO) in 2010s than in 1990s. The warming and salinification of WSIO AABW are primarily explained by changes in source water mixing ratios, although the source water properties also significantly contribute to the observed changes. In contrast, the warming and freshening of ESIO AABW cannot be explained without considering changes in the source water properties as the direction of AABW salinity change due to source water mixing ratios is opposite (salinification) to that of observations (freshening). The east–west contrasting pattern of AABW salinity changes and more rapid warming in the ESIO have important consequences for poleward AABW transport and sea-level rise within and beyond the SIO.

## Introduction

**Figure 1:**
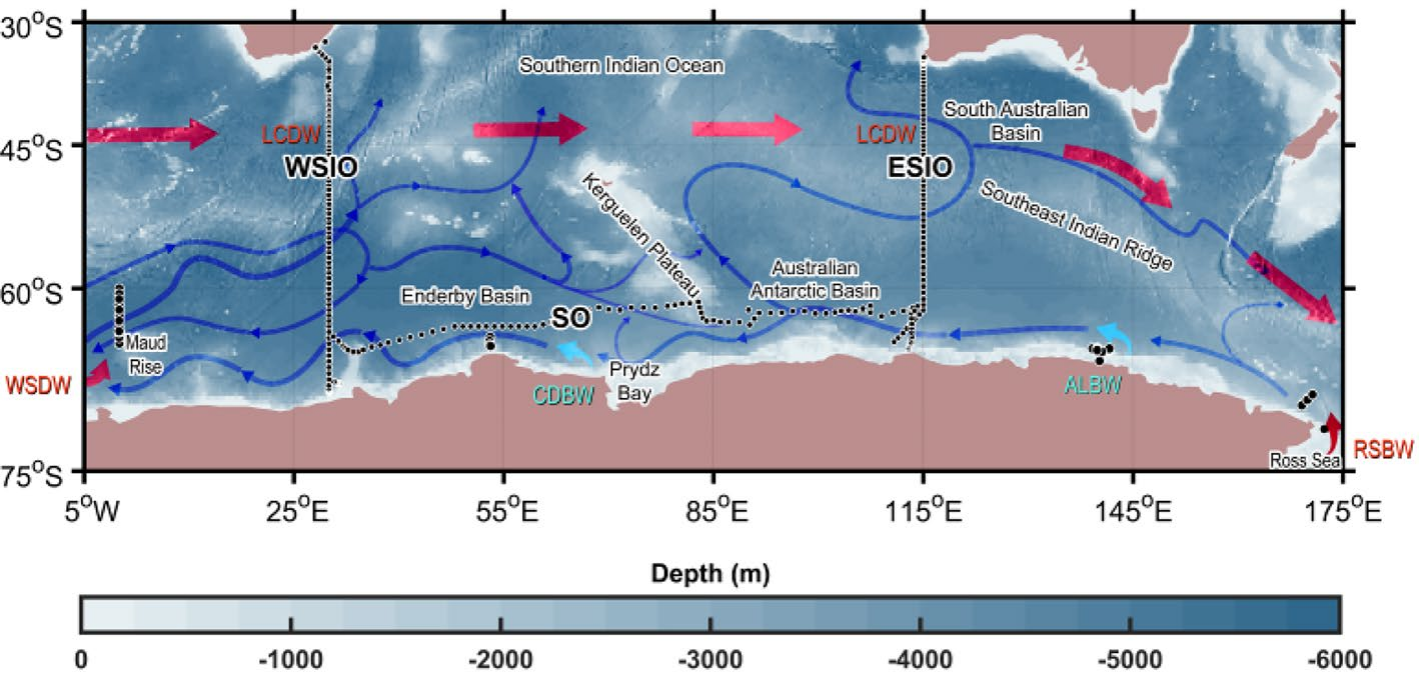
Schematic of the primary sources and paths of Antarctic Bottom Water (AABW) in Southern Indian Ocean (SIO), with ocean depth highlighted. Schematic pathways of AABW (blue dashed arrows) and Lower Circumpolar Deep Water (LCDW; thick red arrows) based on previous works^1,3,5,7–9,13–20,28,30–32^ are superimposed with ocean depth (shaded). Source water formation regions (Weddell Sea Deep Water, WSDW; Cape Darnley Bottom Water, CDBW; Adélie Land Bottom Water, ALBW; and Ross Sea Bottom Water, RSBW) are marked using solid arrows and labels (red for WSDW and RSBW, cyan for CDBW and ALBW). Locations of ship-based hydrographic data collected along the meridional lines in western (WSIO) and eastern SIO (ESIO), and the zonal line (at ~ 65° S) in the Southern Ocean (SO) are marked with black dots.

Antarctic Bottom Water (AABW), generally defined as seawater with a potential temperature (θ) less than 0 °C or neutral density (γ^n^) greater than 28.27 kg m^−3^, forms near the Antarctic continental margins^1–5^. AABW ventilates the abyssal ocean, sequesters heat and carbon, and regulates the global overturning circulation and atmospheric carbon dioxide^4–7^. There are four identified regions of AABW formation; AABW has been reported to have slightly different properties in each of them^4,5,8–14^ (Fig. 1). The properties of AABW in the Indian sector of the Southern Ocean (Southern Indian Ocean; hereafter SIO) are determined by hydrographic conditions in the formation regions with an abyssal circulation primarily constrained by bathymetry and major currents such as the eastward-flowing Antarctic Circumpolar Current (ACC) and westward-flowing Antarctic Slope Current near the Antarctic shelves^3,13–16^. AABW in the western SIO (WSIO) reflects the properties of source waters such as Lower Circumpolar Deep Water (LCDW), Weddell Sea Deep Water (WSDW), and Cape Darnley Bottom Water (CDBW)^17,18^. In contrast, AABW in the eastern SIO (ESIO) consists of LCDW, Adélie Land Bottom Water (ALBW), and Ross Sea Bottom Water (RSBW) (Fig. 1 and Table 1)^19,20^. Anthropogenically emitted greenhouse gases have affected ocean circulation^21,22^ by forcing pattern changes in the Southern Annular Mode (SAM)^23,24^, and consequently, have impacted the properties of AABW and its source waters^25–28^. Continued warming and freshening of AABW in the ESIO have been investigated^25–29^. Increases in θ of 0.02–0.08 °C decade^−1^ and decreases in practical salinity (S_P_) of 0.002–0.012 decade^−1^ have been observed between the 1990s and 2000s^2,5,6,29^. Significant warming (> 0.03 °C decade^−1^) before 2000 and slight salinification (< 0.001 decade^−1^) in the WSIO (near 40°E) between the 1970s and 2010s have also been reported^18,21^. However, despite previous studies on changes in AABW characteristics during recent decades, our understanding of their causes remains at a primitive stage. In this study, we analysed an east–west contrasting salinity change ($\Delta$S_P_) in AABW (defined as θ < 0 °C and γ^n^ > 28.27 kg m^−3^) in addition to overall warming ($\Delta\uptheta$ > 0 °C) in the WSIO and ESIO over the past three decades. Our analysis demonstrates how the properties of and mixing ratios between source waters affect AABW characteristics in global ocean circulation under a changing climate regime.

**Table 1:** Mean time difference (Δt, years), potential temperature (θ, °C), practical salinity (S_P_), and differences and trends (1990s–2010s) of θ and S_P_ for observed (Observation) and estimated (Case 1 and Case 2) Antarctic Bottom Water (AABW) and corresponding source waters in the western SIO (WSIO) and eastern SIO (ESIO). Confidence intervals (95%) are written after each value. Case 1 (Case 2) reproduced the AABW characteristics using time-varying (fixed) mixing ratios between and fixed (time-varying) properties of source waters.

Observation area	Water mass considered	Δt^a^ (year)	θ (°C)		Δθ (°C)	Δθ/Δt (°C decade^−1^)
			1990s	2010s	2010s − 1990s	
WSIO	AABW (Observation)	24.5	− 0.42 ± 0.01	− 0.37 ± 0.01	0.05 ± 0.02	0.02 ± 0.01
	AABW (Case 1)		− 0.40 ± 0.01	− 0.37 ± 0.01	0.03 ± 0.02	0.01 ± 0.01
	AABW (Case 2)		− 0.41 ± 0.01	− 0.39 ± 0.01	0.02 ± 0.02	0.01 ± 0.01
	CDBW	19.7	− 0.57^b^	− 0.53 ± 0.02	0.04 ± 0.02	0.02 ± 0.01
	WSDW	16.0	− 0.68 ± 0.02	− 0.70 ± 0.01	− 0.02 ± 0.03	− 0.01 ± 0.02
	LCDW	24.5	2.03 ± 0.21	2.06^b^	0.03 ± 0.21	0.01 ± 0.09
ESIO	AABW (Observation)	17.0	− 0.18 ± 0.01	− 0.13 ± 0.01	0.05 ± 0.02	0.03 ± 0.01
	AABW (Case 1)		− 0.16 ± 0.01	− 0.13 ± 0.01	0.03 ± 0.02	0.02 ± 0.01
	AABW (Case 2)		− 0.17 ± 0.01	− 0.14 ± 0.01	0.03 ± 0.02	0.02 ± 0.01
	RSBW	23.6	− 0.72 ± 0.04	− 0.53 ± 0.04	0.19 ± 0.04	0.08 ± 0.02
	ALBW	19.2	− 0.78 ± 0.08	− 0.81 ± 0.08	− 0.03 ± 0.16	− 0.01 ± 0.08
	LCDW	17.0	1.75^b^	1.76^b^	0.01^b^	0.01^b^

Observation area	Water mass considered	Δt^a^ (year)	S_P_		ΔS_P_	ΔS_P_/Δt (decade^−1^)
			1990s	2010s	2010s − 1990s	
WSIO	AABW (Observation)	24.5	34.660 ± 0.001	34.664 ± 0.001	0.004 ± 0.002	0.002 ± 0.001
	AABW (Case 1)		34.659 ± 0.001	34.662 ± 0.001	0.003 ± 0.002	0.001 ± 0.001
	AABW (Case 2)		34.661 ± 0.001	34.662 ± 0.001	0.001 ± 0.002	0.000 ± 0.001
	CDBW	19.7	34.644^b^	34.640 ± 0.005	− 0.004 ± 0.005	− 0.002 ± 0.003
	WSDW	16.0	34.652 ± 0.002	34.659 ± 0.001	0.007 ± 0.003	0.004 ± 0.002
	LCDW	24.5	34.819 ± 0.024	34.825^b^	0.006 ± 0.025	0.002 ± 0.010
ESIO	AABW (Observation)	17.0	34.681 ± 0.001	34.673 ± 0.001	− 0.008 ± 0.002	− 0.005 ± 0.001
	AABW (Case 1)		34.673 ± 0.001	34.680 ± 0.001	0.007 ± 0.002	0.004 ± 0.001
	AABW (Case 2)		34.685 ± 0.001	34.670 ± 0.001	− 0.015 ± 0.002	− 0.008 ± 0.001
	RSBW	23.6	34.727 ± 0.025	34.696 ± 0.021	− 0.031 ± 0.046	− 0.013 ± 0.023
	ALBW	19.2	34.640 ± 0.003	34.627 ± 0.003	− 0.013 ± 0.006	− 0.007 ± 0.003
	LCDW	17.0	34.751^b^	34.754^b^	0.003^b^	0.002^b^

^a^Time difference of two observation periods between the 2010s and 1990s, estimated after averaging the observation periods in each decade from the years listed in Supplementary Table 2.

^b^Confidence interval is not available from one sample from a single cruise for the corresponding decade.

## Results

### Warming and salinification of AABW in the WSIO

**Figure 2:**
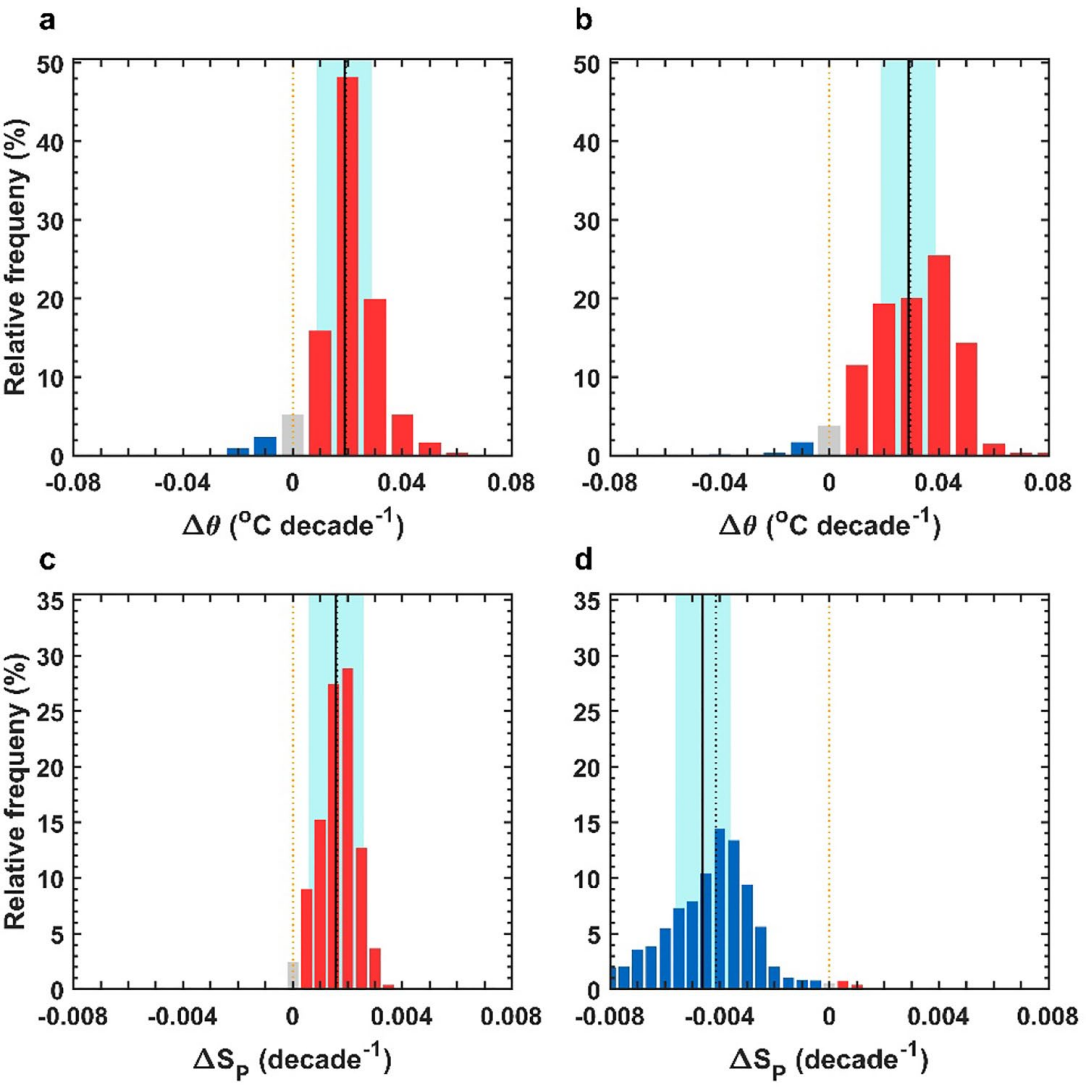
Changes in potential temperature (θ) and practical salinity (S_P_) of Antarctic Bottom Water (AABW) in the Southern Indian Ocean (SIO). Histograms of $\Delta\uptheta$ (**a**, **b**) and $\Delta$ S_P_ (**c**, **d**) of AABW in the western (WSIO; **a**, **c**) and eastern SIO (WSIO; **b**, **d**) between the 1990s and 2010s. Blue (red) indicates cooling and freshening (warming and salinification), whereas grey indicates no change. Areas shaded in cyan represent statistical significance at the 95% confidence interval from the mean, based on Student’s *t*-distribution. The solid black lines indicate mean values, while zero and median values are shown using orange and black vertical dashed lines, respectively.

In the WSIO, we observed that AABW $\uptheta$ increased from − 0.42 °C in the 1990s to − 0.37 °C in the 2010s over 24.5 years ($\Delta \mathrm{t}$), indicating warming ($\Delta\uptheta$ of + 0.05 °C) with a warming rate ($\Delta\uptheta /\Delta \mathrm{t}$) of + 0.02 ± 0.01 °C decade^−1^ (Table 1). Most areas in the AABW domain exhibited significant (95% confidence level) warming between the 1990s and 2010s **(**Fig. 2a and Supplementary Fig. 1a). The observed AABW S_P_ also increased in the WSIO from 34.660 in the 1990s to 34.664 in the 2010s (Table 1). Therefore, salinification occurred ($\Delta$S_P_ of + 0.004) at a salinification rate ($\Delta$S_P_$/\Delta \mathrm{t}$) of + 0.002 ± 0.001 decade^−1^ (Table 1). Significant salinification was found almost everywhere within the WSIO AABW domain between the 1990s and 2010s (Fig. 2c and Supplementary Fig. 1c).

**Figure 3:**
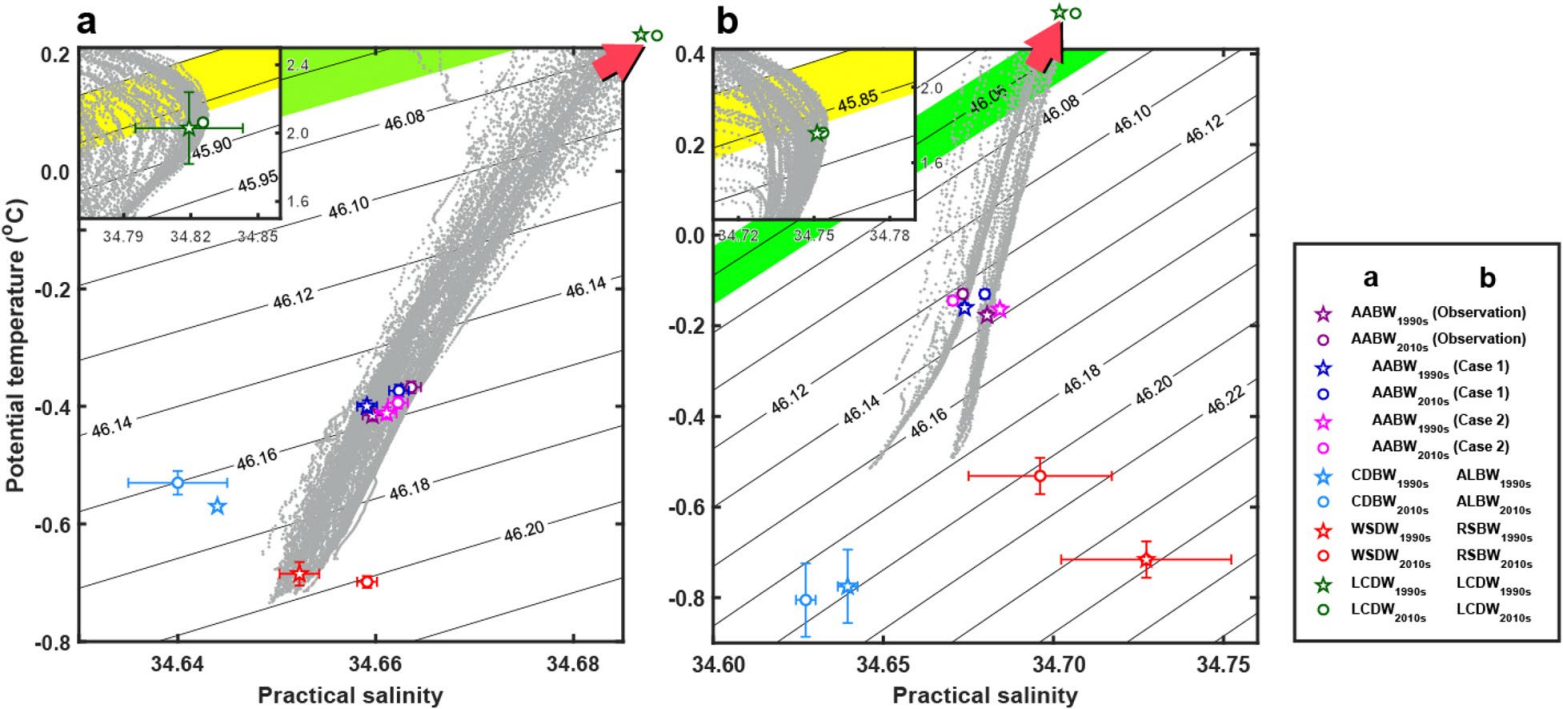
θ–S_P_ plots of Antarctic Bottom Water (AABW) and source waters in Southern Indian Ocean (SIO). θ–S_P_ plots of AABW (purple in **a**, **b**) and estimated (Case 1, blue; Case 2, magenta) in the western (WSIO) (**a**) and eastern SIO (ESIO) (**b**). CDBW (**a**) and ALBW (**b**) are coloured with light blue, LCDW (**a**, **b**) with green, and WSDW (**a**) and RSBW (**b**) with red for the 1990s (asterisks) and 2010s (circles) with error bars for 95% confidence interval (see Table 1). The black contours denote potential density (kg m^−3^) referenced to 4000 dbars (${\sigma }_{4}$). LCDW properties are found only in the zoomed-out domain (top-left corners) and out of the range in the zoomed-in domain, as marked by the red shaded arrows. Neutral densities of 28.05 and 28.27 kg m^−3^, corresponding to 45.80–45.86 kg m^−3^ and 48.05–48.07 kg m^−3^ in ${\sigma }_{4}$, are shaded in thick yellow and light green, respectively. *WSDW* Weddell Sea Deep Water, *CDBW* Cape Darnley Bottom Water, *ALBW* Adélie Land Bottom Water, *RSBW* Ross Sea Bottom Water.

The warming and salinification of AABW in the WSIO were examined with time-varying mixing ratios between source waters with fixed properties (Case 1; see “Methods” section for details) and conversely, with fixed mixing ratios between source waters with time-varying properties (Case 2; see “Methods” section for details). The direction of changes in WSIO AABW characteristics (warming and salinification) can be explained by changes in mixing ratios between the source waters (Case 1), rather than in their properties (Case 2) (Fig. 3a and Table 1). A reduced portion of fresh CDBW (from 60 to 52%) and increased portions of warm and saline LCDW and saline WSDW (from 7 to 9% and 33–38%, respectively) were mainly responsible for the warming and salinification (Supplementary Table 1). Similarly, values estimated based on the time-varying properties and fixed mixing ratios between the source waters (Case 2) were consistent with the observations and indicated a warming trend although salinification was not significant (Fig. 3a and Table 1). CDBW is the largest contributing source water to the WSIO AABW, with the highest (52–60%) mixing ratio between the source waters. The $\uptheta$ of CDBW increased ($\Delta\uptheta$ = 0.04 °C) over the decades though the S_P_ of CDBW decreased ($\Delta$S_P_ =  − 0.004), and WSDW showed salinification at a rate ($\Delta$S_P_$/\Delta \mathrm{t}$) of 0.004 ± 0.002 decade^−1^ (Table 1 and Supplementary Table 1).

Although the WSIO AABW warming that was apparent in our observations was reproduced with both time-varying mixing ratios between the source waters (Case 1) and time-varying source water properties (Case 2), the salinification could be explained only by the former (Case 1). Also, considering only the changes in mixing ratios (Case 1) or only source water properties (Case 2), the warming was underestimated compared to the observations (Table 1). The observed changes (both direction and magnitude) in WSIO AABW characteristics (Δθ and ΔS_P_) can be best explained by considering the changes in mixing ratios between the source waters and their properties. However, WSIO AABW warming and salinification are primarily accounted for by the changes in the mixing ratios between the source waters.

Long-term warming and salinification trends in the WSIO AABW have been previously reported^18,21^. For example, warming and salinification at 0.03 °C decade^−1^ and < 0.001 decade^−1^, respectively, were found between the 1970s and 2010s based on observations in the Enderby Basin^18^ (Fig. 1). Slower warming and more rapid salinification between the 1990s and 2010s were observed compared with those from the more extended period (Table 1). Salinification and the overall warming of deep and bottom waters in the northeast Weddell Sea (reported previously as, e.g., $\Delta$S_P_ of + 0.002 between the early 1990s and 2000s^20,33^) were comparable to the WSDW salinification rate (0.004 decade^−1^) presented in Table 1. This WSDW salinification was partly responsible for AABW salinification. Warming and salinification of deep and bottom waters in the Weddell Sea are potentially linked to the increasing LCDW flow into AABW beyond the WSDW formation region^20^. Considerable LCDW inflow into the Weddell Sea and resultant salinification of WSDW could be related to positive SAM index anomalies over the last decade^20,34^ as Ekman suction driven by anomalously strong westerly winds in the SAM positive phase strengthens the Weddell Gyre and ACC along with overriding the locally formed denser WSDW^20^, thereby increasing transport of highly saline LCDW into the deep and bottom waters of the Weddell Sea.

### Warming and freshening of AABW in the ESIO

As in the WSIO, AABW $\uptheta$ observed along meridional lines in the ESIO also increased from − 0.18 °C in the 1990s to − 0.13 °C in the 2010s ($\Delta \mathrm{t}$ = 17.0 years), yielding a warming rate ($\Delta\uptheta /\Delta \mathrm{t}$) of + 0.03 ± 0.01 °C decade^−1^, that is, 1.5 times higher than that in the WSIO (Table 1). In most of the ESIO AABW domain, significant warming was observed between the 1990s and 2010s, like that in the WSIO (Fig. 2b and Supplementary Fig. 1b). However, S_P_ decreased such that the ESIO AABW freshened, from 34.681 in the 1990s to 34.673 in the 2010s; this contrasted with the WSIO salinification (Table 1). Over the 17 years, $\Delta$S_P_ and $\Delta$S_P_$/\Delta \mathrm{t}$ were − 0.008 and − 0.005 ± 0.001 decade^−1^, respectively. Nearly all of the ESIO AABW domain experienced significant freshening between the 1990s and 2010s (Fig. 2d and Supplementary Fig. 1d).

The warming and freshening of AABW, particularly the direction of changes in AABW characteristics in the ESIO, can be explained by the scenario wherein the source waters have time-varying properties (Case 2) rather than fixed ones (Case 1); the former yielded warming and freshening at slightly lower (e.g., slower warming) and higher (e.g., more rapid freshening) rates than the observations (Fig. 3b and Table 1). S_P_ decreases between the 1990s and 2010s were significant in ALBW and RSBW, whereas significant $\uptheta$ increases were found in RSBW only (Fig. 3b and Table 1). In contrast, changing mixing ratios between the source waters with no change in source water properties (Case 1) could not reproduce the observed freshening as yield salinification (Fig. 3b and Table 1). The mixing ratios of relatively cold and fresh ALBW decreased from 60 to 52%, and those of warm and saline LCDW slightly increased from 23 to 24%, whereas the mixing ratio of the relatively saline RSBW significantly increased from 17 to 24% (Supplementary Table 1). As a result, AABW $\uptheta$ and S_P_ increased over the decades, which is inconsistent with observations (Fig. 3b and Table 1). Thus, the warming and freshening of source waters were responsible for the changes in the ESIO AABW characteristics. The ESIO AABW warming and freshening cannot be explained without considering the change in source water properties. However, the under- and overestimated rates of changes (lower $\Delta\uptheta /\Delta \mathrm{t}$ and higher ΔS_P_/Δt) produced by the changing source water properties (Case 2) can be corrected only by considering the changes in mixing ratios between them (Case 1) as well.

The AABW warming and freshening rates on the west side of the Australian Antarctic Basin (Fig. 1) were previously reported as 0.02–0.08 °C decade^−1^ and 0.002–0.012 decade^−1^, respectively^2,5,6,29^, comparable to the results in this study (Table 1). The warming and freshening of ESIO AABW are related to the overall abyssal warming in the Southern Ocean and the freshening of source waters around the Antarctic shelves. Previous studies suggest that bottom waters in the Southern Ocean have warmed at a rate of ~ 0.05 °C decade^−1^, leading to an increase in ocean heat content^21^, which is consistent with the overall warming of both ESIO and WSIO AABW in this study (Fig. 3 and Table 1). In the ALBW and RSBW formation regions, near-bottom water has experienced remarkable freshening with decreases in absolute salinity of ~ 0.06 g kg^−1^ from the 1990s to early 2010s^19^. These freshening rates are higher than those of the ESIO AABW reported either from observations or reproduction by only considering changes in source water properties (Case 2) (Fig. 3b and Table 1). The AABW freshening in the Southern Ocean might be due to a decrease in sea ice production, continental ice discharge, and abrupt glacial calving events near the ALBW formation region in 2010; warming and freshening have occurred in the ALBW from the 1960s to the 2000s^19,35,36^. In February 2010, an abrupt calving event occurred on the Mertz Glacier near the ALBW formation region. This event might have caused increasing freshwater input while decreasing the scale of polynya activity and sea ice production^37^. Large-scale atmospheric and ocean circulations may affect the warm and saline Upper Circumpolar Deep Water (placed above the LCDW) intrusion onto the continental shelves of the Amundsen and Bellingshausen Seas, and subsequent melting of the western Antarctic ice shelves^38,39^. When the easterlies prevail over the Amundsen Sea, the local polynya activity in the Ross Sea (including the RSBW formation region) can be reduced by causing an inflow of sea ice from the Amundsen Sea^24^. The reduced polynya activity causes a reduction in dense water formation through brine rejection, resulting in decreases in RSBW volume and salinity^15,19^.

Since the late 2010s, however, salinity has rebounded in the ALBW formation region and western Ross Sea^19,40^. Between 2015 and 2018, positive SAM and strong El Niño conditions reduced the strength of easterly winds, which sequentially reduce sea ice input from the Amundsen Sea^24^. These conditions left the Ross Sea more open, which promoted polynya activity and produced a greater amount of highly saline shelf water due to brine rejection^24,41^. This recent salinity increase is closely related to the increase in the S_P_ of High Salinity Shelf Water (~ 0.07 since 2014), a precursor of RSBW salinification^41^, which might cause the salinification of ESIO AABW in the 2020s. Further studies are needed to address the salinity rebound processes in the 2020s and decadal oscillations of AABW characteristics with continued hydrographic observations across the Southern Ocean.

### Implication of contrasting salinity changes in AABW between WSIO and ESIO

The contrasting salinity changes of AABW between the WSIO and ESIO (western salinification and eastern freshening) and the overall SIO warming over the past three decades were analysed based on changes in the mixing ratio between (Case 1) and properties (Case 2) of source waters. We found that both these changes were important causes of the western salinification and eastern freshening at the observed rates. However, the directions of changes in AABW salinity are primarily caused by increasing (decreasing) ratios of relatively saline (fresh) source waters in the WSIO, e.g., higher mixing ratios of WSDW and LCDW (lower mixing ratios of CDBW) in the 2010s than the 1990s, and by decreasing salinities of source waters in the ESIO. Regarding the mixing ratios of AABW source waters in the WSIO, the observed AABW characteristics are better explained by including the remotely induced warming from the CDBW, which has sometimes been excluded in previous studies. The ratio of CDBW, ranging from 52 to 60%, is comparable to previously reported values^32^ (60%) despite the overall warm and saline properties of CDBW ($\uptheta$ and S_P_ ranging from − 0.57 to − 0.53 °C and from 34.640 to 34.644, respectively) compared to those defined for a slightly different region (e.g., further upstream, between 65° E and 69° E) in the previous study ($\uptheta$ = − 0.65 °C and S_P_ = 34.635)^9,32^.

**Figure 4:**
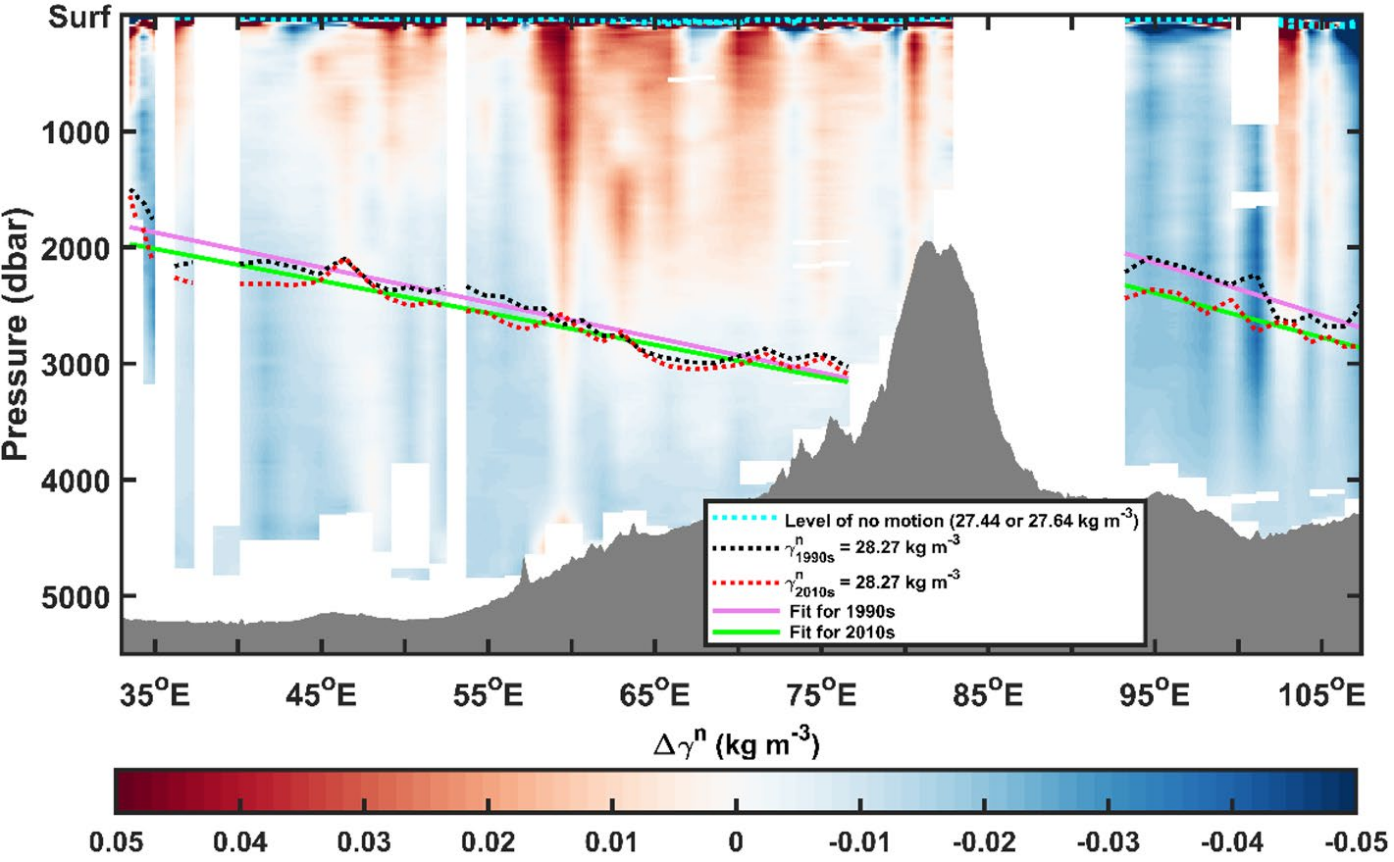
Zonal section of neutral density (γ^n^) change between the 1990s and 2010s with isopycnal surface γ^n^ = 28.27 kg m^−3^ as an upper boundary of AABW across the SO. Cross-sectional structure of neutral density change between the 1990s and 2010s (2010s minus 1990s) where isopycnal of γ^n^ = 28.27 kg m^−3^ in each period are denoted by dashed and solid lines (1990s; black dashed and solid magenta lines, and 2010s; red dashed and solid green lines) superimposed by bottom topography (grey shading area) from the Smith–Sandwell bathymetry data^42^. The cyan dashed lines denote a level of no motion corresponding to isopycnals of γ^n^ = 27.44 kg m^−3^ in the WSIO and γ^n^ = 27.64 kg m^−3^ in the ESIO, respectively.

The contrasting salinity changes and overall warming with different warming rates between WSIO and ESIO (western salinification-eastern freshening and more rapid warming in the ESIO than WSIO; Table 1) may have affected the zonal difference in the AABW density and volume. Previous studies on similar east–west contrasting salinity changes (more rapid freshening in the ESIO and slight salinification in the WSIO) between the 1980s and 2000s assessed the relative contributions of water-mass salinity changes and isotherm heave on the total salinity change, raising the possibility of more heave contributions to the salinification in the WSIO and a greater contribution of water-mass salinity changes to the freshening in the ESIO^6^. The net salinity and temperature effects on the AABW density and volume make the total steric height (net thermosteric and halosteric effects) higher in the ESIO than WSIO (Supplementary Fig. 2). Consequently, the abyssal circulation was affected, as the AABW upper boundary (e.g., the surface of γ^n^ = 28.27 kg m^−3^) deepened eastward more in the 2010s than in the 1990s, decreasing the AABW volume more so in the ESIO than in the WSIO (Fig. 4 and Supplementary Fig. 1). The AABW upper boundary sloped eastward regardless of the period, based on the cross-sectional structure of neutral density and zonal profiles of the surface of γ^n^ = 28.27 kg m^−3^ across the zonal observational line (SO) in the SIO, corresponding to geostrophic abyssal flow toward the Indian Ocean at both the Enderby Basin (WSIO) and Australian Antarctic Basin (ESIO), separated by the Kerguelen Plateau, with a steeper slope in the ESIO (Fig. 4). The steeper slope and correspondingly stronger vertical shear of equatorward geostrophic current toward the Indian Ocean in the ESIO significantly decreased from 1.4 cm s^−1^ in the 1990s to 1.2 cm s^−1^ in the 2010s, referenced to the assumed level of no motion. In contrast, the change in gentler slope and weaker geostrophic flow (~ 0.9 cm s^−1^ in the 1990s and 2010s) toward the Indian Ocean in the WSIO, referenced to the assumed level of no motion, was not significant.

Compared to the 1990s, the deep and abyssal flow toward the Indian Ocean demonstrated a weakening eastern intensification in 2010s. In particular, the zonally contrasting pattern of the changes observed in the AABW characteristics, of which the geostrophic flow speed was similar to that reported in previous observations (typically a few centimetres per second, up to 0.1 m s^−1^)^16,18^, may impact the deep/abyssal and global overturning circulation within and beyond the Indian sector.

The contrasting changes in AABW salinity and overall warming with different warming rates between the WSIO and ESIO also have important implications for global sea-level rise. Previously estimated steric sea-level rises below 3000 dbar in the SIO were 0.01–0.04 m between the 1990s and 2000s and 0.04–0.07 m between the 1990s and 2010s^2,5^. This study estimated a reasonable range of steric sea-level changes relative to 3000 dbar of up to 0.01–0.02 m in WSIO and ESIO, but for different reasons. In the WSIO, thermosteric sea-level rise of ~ 0.02 m was partly compensated by a halosteric sea-level drop of ~ 0.01 m, which yields a relatively small total steric sea-level rise of ~ 0.01 m (Supplementary Fig. 2a,c,e). In contrast, a thermosteric sea-level rise of ~ 0.012 m was reinforced by a halosteric sea-level rise of ~ 0.007 m, yielding a higher total steric sea-level rise of ~ 0.02 m in the ESIO (Supplementary Fig. 2b,d,f). This indicates that the east–west contrasting pattern (western salinification and eastern freshening) of the changes in AABW salinity between the 1990s and 2010s has significant consequences for steric sea-level rise within and beyond the SIO.

## Methods

### Data processing

All high-quality hydrographic Conductivity-Temperature-Depth (CTD) data used in this study were provided by the CLIVAR Carbon Hydrographic Data Office website (https://cchdo.ucsd.edu/) after processing to correct biases and errors. The data processing included salinity correction where the data were calibrated against in situ bottle seawater samples collected along with the CTD data during the cruises and measured using an Autosal salinometer referenced to Standard Sea Waters of the International Association for the Physical Sciences of the Oceans. Following the method described in previous studies, batch-to-batch correction was applied to the CTD data (see also Supplementary Table 3)^6,32,43,44^. To facilitate the comparison between the observed data with those of previous studies, in situ temperature and practical salinity (S_P_) were used to convert potential temperature (θ), potential density reference pressure of 4000 dbar (${\sigma }_{4}$), and neutral density (γ^n^)^45^ using a formula in the Gibbs Seawater Oceanographic Toolbox v3.06, which contains the Thermodynamic Equation of Seawater 2010 (TEOS-10; http://www.teos-10.org/), and the PreTEOS-10 (http://www.teos-10.org/preteos10_software/). Noise was removed from the CTD data (including near bottom data) using a moving average over a 20-dbar interval and then interpolated to 0.1° × 10-dbar intervals through linear interpolation. To estimate the uncertainties of θ and S_P_ of AABW and source waters, a 95% confidence interval from Student’s *t*-distribution was used, as given by $\overline{x}\pm {t }_{{\alpha /2}_{v}}\frac{s}{\sqrt{v}}$, where $\overline{x }$, $s$, $v$, and ${t}_{{\alpha /2}_{v}}$ represent the mean and standard deviation of the samples collected in different time and spaces (varying over time as well as space), effective degrees of freedom, and critical value of the *t* statistic of $v$, respectively^5^. We assumed that uncertainties in the mean temperature and salinity did not vary across seasons but by the mean itself, applying temporal decorrelation scale of one year. A spatial decorrelation length scale of 160 km, used in a previous study, was applied to estimate the effective degrees of freedom^5^.

### Determination of AABW and source water properties

The CTD data collected along the meridional observational lines in 1993, 1996, and 2019 in the WSIO (Fig. 1), 1995 and 2012 in the ESIO (Fig. 1), and along the zonal observational lines (at ~ 65°S) in 1996 and 2013 off the Antarctic shelves (SO, Fig. 1) were used to analyse AABW characteristics and their changes between the 1990s and 2010s (Supplementary Table 2). The AABW domain was defined where γ^n^ > 28.27 kg m^−3^ and θ < 0 °C to estimate the θ and S_P_ differences in AABW between the 1990s and 2010s.

The endmembers of source waters were estimated from CTD data based on previous studies^9,17–20^ (Supplementary Table 2). In the WSIO, endmembers of three source waters (WSDW, CDBW, LCDW) were determined by averaging θ and S_P_ over the following areas: − 0.7 °C < θ < 0 °C within 60°–65° S and 0.5° W–0.5° E with a maximum depth > 2500 dbar for WSDW, 64–70° S and 50–61° E with a maximum depth > 1000 dbar for CDBW, and maximum salinity of 28.05 < γ^n^ < 28.27 kg m^−3^ for LCDW^18,20^ (Table 1, Supplementary Table 1). In the ESIO, endmembers of three source waters (RSBW, ALBW, and LCDW) were determined by averaging θ and S_P_ over the following areas: For RSBW, 69.2–78° S and 160–180° E with a maximum depth > 1300 dbar, 65.3–66.0° S and 138–144° E with a maximum depth > 400 dbar for ALBW, and maximum salinity in 28.05 < γ^n^ < 28.27 kg m^−3^ for LCDW (Table 1, Supplementary Table 1)^9,17,19^.

Since some source water properties (θ and S_P_ of CDBW, and θ of ALBW) significantly varied across seasons, we removed the seasonal θ and S_P_ variations inferred from previously reported mooring data^9,11^ from the cruise CTD data collected in the study areas in different years and seasons when it was available. Due to the lack of long and continuous observations, one year-long time series observations reported in previous studies^9,11^ were used for seasonal climatology, inevitably limiting the approach. First, the source water properties observed during the cruise season within the area were spatially averaged ((1) in Supplementary Table 4). Then, the anomalies ((4) in Supplementary Table 4) obtained by removing monthly seasonal mean properties derived from mooring data (by 3-month moving-average) ((2) in Supplementary Table 4) were added to the mean properties of October (September–October–November average) ((3) in Supplementary Table 4). For the case S_P_, bias in the anomalies ((5) in Supplementary Table 4) was further shifted to correct spatial difference between the mooring location and source water area ((7) in Supplementary Table 4). Here, our assumption on the most effective contribution of the source water into the AABW formation during September–October–November is supported by the mooring observations, e.g., density, near-bottom layer thickness, and downslope transport reach their maxima, although those in other months are non-zero.

### Reproducing AABW temperature and salinity

Characteristics of AABW were reproduced through a simple optimum multiparameter analysis using two conservative source water tracers (θ and S_P_)^17^.

1a $${x}_{1}{\theta }_{1}+{x}_{2}{\theta }_{2}+{x}_{3}{\theta }_{3}= {\theta }_{AABW}$$

1b $${x}_{1}{S}_{1}+{x}_{2}{S}_{2}+{x}_{3}{S}_{3}= {S}_{AABW}$$

1c $${x}_{1}+{x}_{2}+{x}_{3}=1$$

Equations (1a)–(1c) represent heat, salt, and mass conservation, respectively. θ_1_–θ_3_ and S_1_–S_3_ denotes the endmembers of source waters (θ and S_P_), determined from observations, and θ_AABW_ and S_AABW_ indicate the θ and S_P_ of AABW (Table 1 and Supplementary Table 1). x_1_ to x_3_ are the mixing ratios of source waters derived from solving simultaneous Eqs. (1a)–(1c) by substituting endmembers of source waters (θ_1_ to θ_3_, S_1_ to S_3_) and AABW properties (θ_AABW_, S_AABW_) as input values, which were arranged in an explicit form as Eqs. (2a)–(2d).

2a $${x}_{1}= \frac{1}{D}\times \{{{\theta }_{AABW}}\left({S}_{2}-{S}_{3}\right)+{S}_{AABW}\left({\theta }_{3}-{\theta }_{2}\right)+{\theta }_{2}{S}_{3}-{\theta }_{3}{S}_{2}\}$$

2b $${x}_{2}= \frac{1}{D}\times \{{{\theta }_{AABW}}\left({S}_{3}-{S}_{1}\right)+{S}_{AABW}\left({\theta }_{1}-{\theta }_{3}\right)+{\theta }_{3}{S}_{1}-{\theta }_{1}{S}_{3}\}$$

2c $${x}_{3}= \frac{1}{D}\times \{{{\theta }_{AABW}}\left({S}_{1}-{S}_{2}\right)+{S}_{AABW}\left({\theta }_{2}-{\theta }_{1}\right)+{\theta }_{1}{S}_{2}-{\theta }_{2}{S}_{1}\}$$

2d $$D= {\theta }_{1}\left({S}_{2}-{S}_{3}\right)+{\theta }_{2}\left({S}_{3}-{S}_{1}\right)+{\theta }_{3}({S}_{1}-{S}_{2})$$

The input parameters of endmember properties of source waters and AABW properties in the 1990s, 2010s, and both periods (average), and output variables of mixing ratios used in this study are listed in Supplementary Table 1. To quantify how much the effects of changing mixing ratios between the source waters (Case 1) on the observed changes in AABW characteristics, mixing ratios between three source waters in the 1990s and 2010s were calculated using the endmember properties of source waters averaged over both periods (input c in Supplementary Table 1) and AABW properties in the 1990s and 2010s (inputs a and b in Supplementary Table 1). The effects of changing source water properties (Case 2) on the observed changes in AABW characteristics were quantified using the endmember properties of source waters in the 1990s and 2010s (inputs a and b in Supplementary Table 1) and AABW properties averaged over both periods (input c in Supplementary Table 1).

### Calculating geostrophic velocity and steric sea-level changes

Geostrophic velocity was calculated through the isopycnic slope assuming a three-layered system using the zonal section CTD data (SO). The smallest zonal isopycnal slopes in the WSIO (γ^n^ = 27.44 kg m^−3^) and ESIO (γ^n^ = 27.64 kg m^−3^) corresponding to the interface between the upper-most and middle layers were regarded as the level of no motion (assuming the flow velocity at the middle was zero; v_1_ = 0). The geostrophic balance in the three-layered ocean was calculated as follows,

3 $${{{\varvec{\rho}}}_{2}{\varvec{f}}{\varvec{v}}}_{2}=\frac{({{\varvec{\rho}}}_{1}-{{\varvec{\rho}}}_{0}){\varvec{g}}\Delta {{\varvec{z}}}_{1}}{\Delta {\varvec{x}}}+\frac{({{\varvec{\rho}}}_{2}-{{\varvec{\rho}}}_{1}){\varvec{g}}\Delta {{\varvec{z}}}_{2}}{\Delta {\varvec{x}}}$$

where ${{\varvec{v}}}_{2}$, ${{\varvec{\rho}}}_{0}$, ${{\varvec{\rho}}}_{1}$**,** and ${{\varvec{\rho}}}_{2}$ denote the geostrophic velocity at the lower layer, reference or upper-layer density (1027.36 kg m^−3^ for both WSIO and ESIO), middle-layer density (1028.12 kg m^−3^ for WSIO and 1028.13 kg m^−3^ for ESIO), and lower-layer density (1028.31 kg m^−3^ for WSIO and 1028.31 kg m^−3^ for ESIO), respectively. $\Delta {\varvec{x}}$ means zonal distance across the section (set to 2294 km for WSIO and 713 km for ESIO). $\Delta {{\varvec{z}}}_{1}$ and $\Delta {{\varvec{z}}}_{2}$ indicate zonal difference in the thickness of middle and lower layers, respectively, where the interface is defined by γ^n^ = 28.27 kg m^−3^. ${\varvec{g}}$ and ${\varvec{f}}$ denote acceleration due to gravity and the Coriolis parameter in the given latitude.

Steric sea-level changes relative to 3000 dbars were calculated to estimate the individual contributions of $\theta$ and S_P_ changes in a fashion similar to that used by previous studies^2,5^.

## Supplementary Information

Supplementary Information.

## Data Availability

All repeated hydrographic data used in this study are available through the CLIVAR and Carbon Hydrographic Data Office website (http://cchdo.ucsd.edu). Topography data were provided by the National Oceanic and Atmospheric Administration (NOAA) (https://www.ngdc.noaa.gov/mgg/global/global.html). Combination of shipboard depth soundings and estimated depths based on gravity measurements data^42^ used in this study are available through the U.S. Geological Survey (USGC; https://www.usgs.gov/). All data needed to draw the conclusions in the paper are present in the paper and/or as supplementary information. Additional data related to this paper may be requested from the authors.

## Code availability

MATLAB scripts used for the analyses described in this study can be obtained from the corresponding author upon request.
