# Author Correction: East–west contrasting changes in Southern Indian Ocean Antarctic Bottom Water salinity over three decades

**DOI:** 10.1038/s41598-023-38908-x

**Published:** 2023-07-20

**Authors:** Yeon Choi, SungHyun Nam

**Affiliations:** 1 School of Earth and Environmental Sciences, College of Natural Sciences, Seoul National University, Seoul, 08826 Republic of Korea; 2 Research Institute of Oceanography, College of Natural Sciences, Seoul National University, Seoul, 08826 Republic of Korea

Correction to: *Scientific Reports* https://doi.org/10.1038/s41598-022-16331-y, published online 16 July 2022

The original version of this Article contained errors in Equation 2, sections 2a to 2d, where

2a $${x}_{1}= \frac{1}{D}\times \{{{\theta }_{AABW}\theta }_{1}\left({S}_{2}-{S}_{3}\right)+{S}_{AABW}{S}_{1}\left({\theta }_{2}-{\theta }_{3}\right)+{\theta }_{2}{S}_{3}-{\theta }_{3}{S}_{2}\}$$

2b $${x}_{1}= \frac{1}{D}\times \{{{\theta }_{AABW}\theta }_{1}\left({S}_{2}-{S}_{3}\right)+{S}_{AABW}{S}_{1}\left({\theta }_{2}-{\theta }_{3}\right)+{\theta }_{2}{S}_{3}-{\theta }_{3}{S}_{2}\}$$

2c $${x}_{3}= \frac{1}{D}\times \left\{{{\theta }_{AABW}\theta }_{3}\left({S}_{1}-{S}_{2}\right)+{S}_{AABW}{S}_{3}\left({\theta }_{1}-{\theta }_{2}\right)+{\theta }_{1}{S}_{2}-{\theta }_{2}{S}_{1}\right\}$$

2d $$D= {\theta }_{1}\left({S}_{2}-{S}_{3}\right)+{\theta }_{2}\left({S}_{1}-{S}_{3}\right)+{\theta }_{3}({S}_{1}-{S}_{2})$$

now reads:

2a $${x}_{1}= \frac{1}{D}\times \{{\theta }_{AABW}\left({S}_{2}-{S}_{3}\right)+{S}_{AABW}\left({\theta }_{3}-{\theta }_{2}\right)+{\theta }_{2}{S}_{3}-{\theta }_{3}{S}_{2}\}$$

2b $${x}_{2}= \frac{1}{D}\times \{{\theta }_{AABW}\left({S}_{3}-{S}_{1}\right)+{S}_{AABW}\left({\theta }_{1}-{\theta }_{3}\right)+{\theta }_{3}{S}_{1}-{\theta }_{1}{S}_{3}\}$$

2c $${x}_{3}= \frac{1}{D}\times \{{\theta }_{AABW}\left({S}_{1}-{S}_{2}\right)+{S}_{AABW}\left({\theta }_{2}-{\theta }_{1}\right)+{\theta }_{1}{S}_{2}-{\theta }_{2}{S}_{1}\}$$

2d $$D= {\theta }_{1}\left({S}_{2}-{S}_{3}\right)+{\theta }_{2}\left({S}_{3}-{S}_{1}\right)+{\theta }_{3}({S}_{1}-{S}_{2})$$

The original Article has been corrected.

