# Supplementary material for: East–west contrasting changes in Southern Indian Ocean Antarctic Bottom Water salinity over three decades
Source: Sci Rep. 2022 Jul 16;12:12175. doi: 10.1038/s41598-022-16331-y (PMC9288450; doi:10.1038/s41598-022-16331-y)
Supplement: Supplementary file 1 — Supplementary Information. [file 41598_2022_16331_MOESM1_ESM.docx]

**Supplementary information**

**East-west contrasting changes in Southern Indian Ocean Antarctic Bottom Water salinity over three decades**

Yeon Choi^1^ and SungHyun Nam^1,2^*

^1^ School of Earth and Environmental Sciences, College of Natural Sciences, Seoul National University, Seoul 08826, Republic of Korea

^2^ Research Institute of Oceanography, College of Natural Sciences, Seoul National University, Seoul 08826, Republic of Korea

*Corresponding author

Phone: +82-2-880-4138, Fax: +82-2-871-3269, E-mail: [namsh@snu.ac.kr](mailto:namsh@snu.ac.kr)


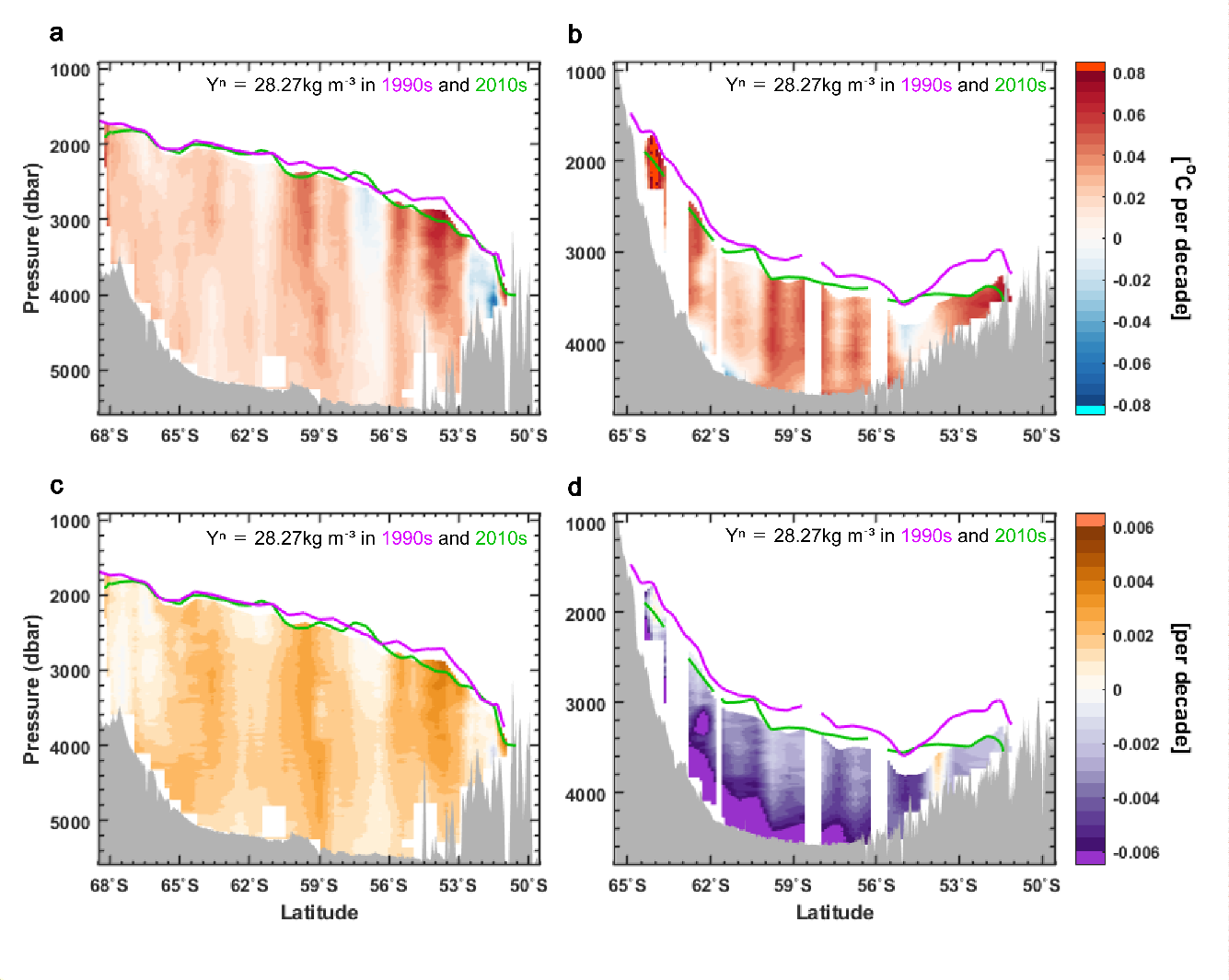


**Supplementary Figure 1.** **Rates of changes in potential temperature and practical salinity**. Rates of changes in potential temperature (°C decade^−1^) and salinity (decade^−1^) in the western (WSIO, **a** and **c**), and eastern Southern Indian Ocean (ESIO, **b** and **d**). Magenta and green lines indicate isopycnals of γ^n^ = 28.27 kg m^−3^ ($\sigma_{4}$; 46.05–46.07 kg m^−3^) in the 1990s and 2010s, respectively. The coloured domain is defined as the area where θ < 0 °C and γ^n^ > 28.27 kg m^−3^ in the entire study period for WSIO and ESIO. Grey shaded area shows bathymetry based on the Smith–Sandwell bathymetry data^[38]^.


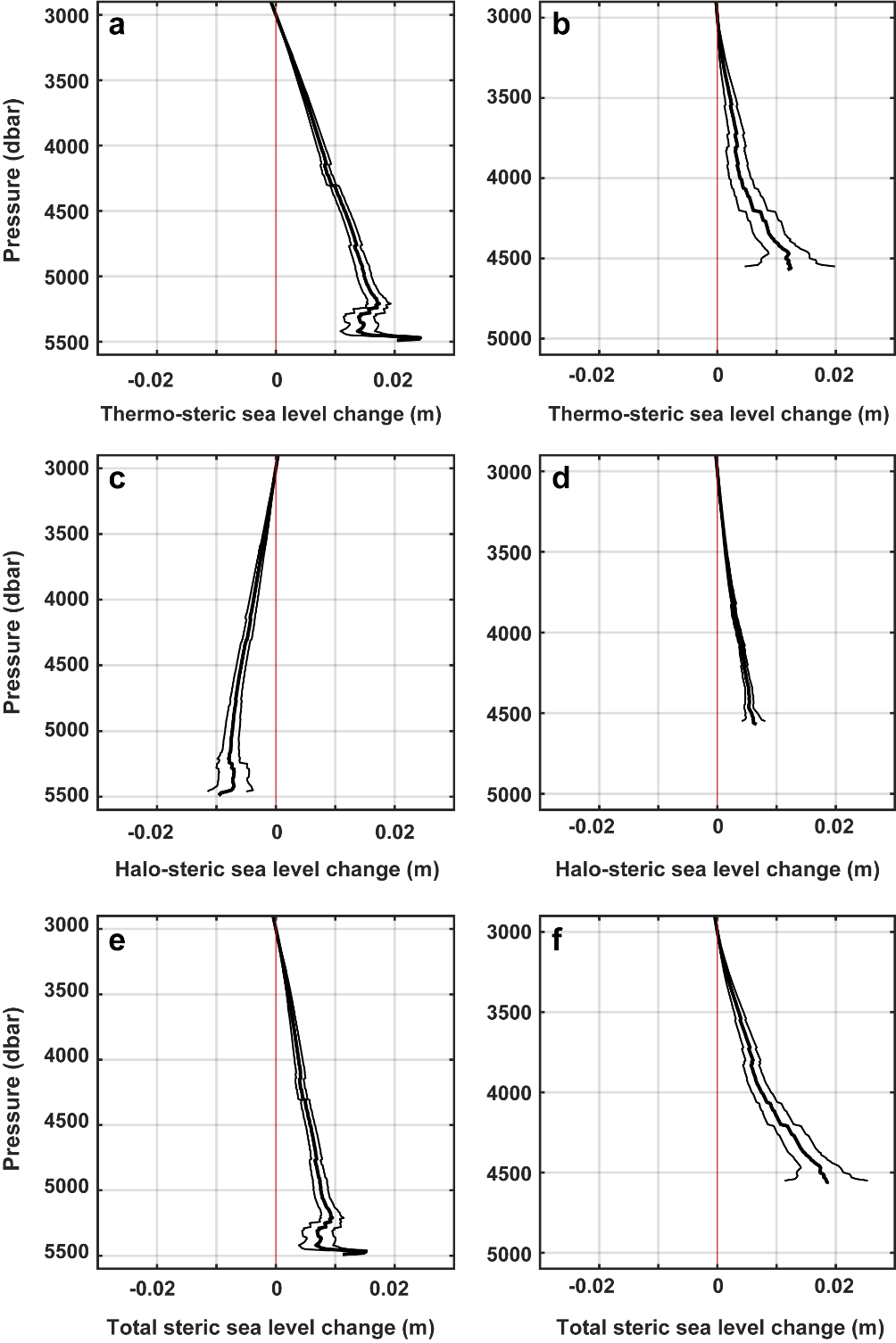


Supplementary Figure 2. Steric sea-level changes relative to 3,000 dbar between the 1990s and 2010s in the WSIO and ESIO. Both thermo- and halo-steric sea-level changes (m) and their sum relative to 3,000 dbar (thick black lines) with 95% confidence limits (thin black lines) between 1990s and 2010s in the WSIO (a, c, and e) and ESIO (b, d, and f) which consider the impacts of temperature (a and b), salinity (c and d) and both effects (e and f).

**Supplementary Table 1. Properties (input θ and S_P_) and mixing ratios (output x in %) with 95% confidence intervals for Cases 1 and 2.** Source water properties constituting the WSIO and ESIO AABW characteristics and observed AABW properties in the (a) 2010s, (b) 1990s, and (c) both periods (on average) as inputs. Mixing ratios between the source waters in the (a) 2010s, (b) 1990s, and (c) both periods (on average) as outputs. The AABW characteristics were reproduced using values in bold (Case 1) or underlined (Case 2).

|  | | | **Input: AABW properties (θ in ^°^C, S_P_)** | **Input: Source water properties (θ in ^°^C, S_P_)** | | | **Output: Mixing ratio (%)** | | |
| --- | --- | --- | --- | --- | --- | --- | --- | --- | --- |
| **WSIO** |  | | **AABW^†^** | **CDBW** ^•^ | **WSDW** ^••^ | **LCDW** ^•••^ | **CDBW** | **WSDW** | **LCDW** |
|  | **a** | **2010s** | −0.37 $\pm$ 0.01,  34.664$\pm$ 0.001 | −0.53 $\pm$ 0.02,  34.640$\pm$ 0.006 | −0.70 $\pm$ 0.01,  34.659 $\pm$ 0.001 | 2.06,  34.825 | 52 ± 4 | 38 ± 4 | 9 ± 1 |
|  | **b** | **1990s** | −0.42 $\pm$ 0.01,  34.660$\pm$ 0.001 | −0.57,  34.644 | −0.68 $\pm$ 0.02,  34.652 $\pm$ 0.002 | 2.03 $\pm$ 0.21,  34.819 $\pm$ 0.024 | 60 ± 4 | 33 ± 4 | 7 ± 1 |
|  | **c** | **All time average** | −0.39 $\pm$ 0.01,  34.662$\pm$ 0.001 | −0.54 $\pm$ 0.01,  34.641$\pm$ 0.006 | −0.69 $\pm$ 0.01,  34.655 $\pm$ 0.008 | 2.04 $\pm$ 0.01  34.821 $\pm$ 0.002 | 52 $\pm4$ | 40 ± 4 | 8 ± 1 |
| **ESIO** |  | | **AABW^†^** | **ALBW^††^** | **RSBW^†††^** | **LCDW** ^•••^ | **ALBW** | **RSBW** | **LCDW** |
|  | **a** | **2010s** | −0.13 $\pm$ 0.01,  34.673$\pm$ 0.001 | −0.81 $\pm$ 0.08,  34.627 $\pm$ 0.002 | −0.53 $\pm$ 0.04,  34.696 $\pm$ 0.021 | 1.76,  34.754 | 52 ± 3 | 24 ± 5 | 24 ± 2 |
|  | **b** | **1990s** | −0.18 $\pm$ 0.01,  34.681$\pm$ 0.001 | −0.78 $\pm$ 0.08,  34.640 $\pm$ 0.003 | −0.72$\pm$ 0.04,  34.727 $\pm$ 0.025 | 1.75,  34.751 | 60 ± 3 | 17 ± 5 | 23 ± 2 |
|  | **c** | **All time average** | −0.15 $\pm$ 0.01,  34.677$\pm$ 0.001 | −0.79 $\pm$ 0.09,  34.635 $\pm$ 0.008 | −0.58 $\pm$ 0.13,  34.704 $\pm$ 0.027 | 1.76 $\pm$ 0.01,  34.752$\pm$ 0.005 | 56 ± 4 | 20 ± 6 | 24 ± 3 |

Abbreviations: AABW, Antarctic Bottom Water; ALBW, Adélie Land Bottom Water; RSBW, Ross Sea Bottom Water; CDBW, Cape Darnley Bottom Water; WSDW, Weddell Sea Deep Water; LCDW, Lower Circumpolar Deep Water

**Supplementary Table 2. Summary of ship-based hydrographic data used in this study**

| Water of concern | Section | Period | Year | Mean year | Research vessel | Cruise designator | Publication  (if available) |
| --- | --- | --- | --- | --- | --- | --- | --- |
| AABW* | WSIO | 1990s | 1993 | 1994.5 | R/V Marion | 35MFCIVA_1 | [25, 26] |
|  |  |  | 1996 |  | R/V Marion | 35MF103_1 | [25, 26] |
|  |  | 2010s | 2019 | 2019.0 | R/V Thomas G. Thompson | 325020190403 |  |
|  | ESIO | 1990s | 1995 | 1995.0 | R/V Knorr | 316N145_5 | [25, 26] |
|  |  | 2010s | 2012 | 2012.0 | R/V Aurora Australis | 09AR20120105 | [19] |
|  | SO | 1990s | 1996 | 1996.0 | R/V Nathaniel B. Palmer | 320696_3 |  |
|  |  | 2010s | 2013 | 2013.0 | R/V Mirai | 49NZ20130106 |  |
| Water of concern | **Section** | **Period** | **YYYYMM** | **Mean year** | **Research Vessel** | **Cruise designator** | **Publication**  **(if available)** |
| LCDW^§^ | WSIO | 1990s | 199302 | 1994.7 | R/V Marion | 35MFCIVA_1 | [25, 26] |
|  |  |  | 199603 |  | R/V Marion | 35MF103_1 | [25, 26] |
|  |  | 2010s | 201904 | 2019.3 | R/V Thomas G. Thompson | 325020190403 | [25, 26] |
|  | ESIO | 1990s | 199501 | 1995.1 | R/V Knorr | 316N145_5 | [25, 26] |
|  |  | 2010s | 201201 | 2012.1 | R/V Aurora Australis | 09AR20120105 | [19] |
| CDBW^§§^ | WSIO | 1990s | 199605 | 1996.4 | R/V Nathaniel B. Palmer | 320696_3 |  |
|  |  | 2010s | 201301 | 2016.1 | R/V Mirai | 49NZ20130106 |  |
|  |  |  | 201901 |  | R/V Mirai | 49NZ20191229 |  |
| WSDW^§§§^ |  | 1990s | 199206 | 1996.0 | R/V Polarstern | 06AQANTX_4 | [30] |
|  |  |  | 199604 |  | RV Polarstern | 06AQANTXIII_4 | [30] |
|  |  |  | 199903 |  | R/V Polarstern | 06AQ199901_2 | [30] |
|  |  | 2010s | 201012 | 2012.0 | R/V Polarstern | 06AQ20101128 |  |
|  |  |  | 201412 |  | R/V Polarstern | 06AQ20141202 |  |
| ALBW^†^ | ESIO | 1990s | 199401 | 1995.3 | R/V Aurora Australis | 09AR9407_1 | [19] |
|  |  |  | 199501 |  | R/V Aurora Australis | 09AR9404_1 | [19] |
|  |  |  | 199603 |  | R/V Aurora Australis | 09AR9604_1 | [19] |
|  |  |  | 199608 |  | R/V Aurora Australis | 09AR9601_1 |  |
|  |  | 2010s | 201101 | 2014.5 | R/V Aurora Australis | 09AR20110104 | [19] |
|  |  |  | 201801 |  | R/V Investigator | 096U20180111 | [19] |
| RSBW^††^ |  | 1990s | 199203 | 1992.0 | R/V Akademik Ioffe | 90KDIOFFE6_1 | [19] |
|  |  | 2010s | 201102 | 2015.6 | R/V Nathaniel B. Palmer | 320620110219 | [19] |
|  |  |  | 201704 |  | R/V Nathaniel B. Palmer | 320620170410 |  |
|  |  |  | 201803 |  | R/V Nathaniel B. Palmer | 320620180309 | [19] |

Abbreviations: AABW, Antarctic Bottom Water; ALBW, Adélie Land Bottom Water; RSBW, Ross Sea Bottom Water; CDBW, Cape Darnley Bottom Water; WSDW, Weddell Sea Deep Water; LCDW, Lower Circumpolar Deep Water

**Supplementary Table 3. Table for batch number of International Association for the Physical Sciences of the Oceans standard seawater (SSW) with recommended salinity offset**^[42,43]^ **used in this study.** Batch offset was applied to most data except for those for which the batch number could not be confirmed through the cruise report or previous reports^[6]^.

Abbreviations: AABW, Antarctic Bottom Water; ALBW, Adélie Land Bottom Water; RSBW, Ross Sea Bottom Water; CDBW, Cape Darnley Bottom Water; WSDW, Weddell Sea Deep Water; LCDW, Lower Circumpolar Deep Water

| Water of concern | Section | Year | Batch  number | SSW offset  ($\boldsymbol{\times}$10^3^) | Cruise designator |
| --- | --- | --- | --- | --- | --- |
| AABW* | WSIO | 1993 | P121  - | 0.4 | 35MFCIVA_1 |
|  |  | 1996 |  |  | 35MF103_1 |
|  |  | 2019 | P162 | −0.5 | 325020190403 |
|  | ESIO | 1995 | P128 | 1.4 | 316N145_5 |
|  |  | 2012 | P153 | 0.4 | 09AR20120105 |
|  | SO | 1996 | P125 | 0.2 | 320696_3 |
|  |  | 2013 | P154 | 0.5 | 49NZ20130106 |
| LCDW^§^ | WSIO | 1993 | P121  - | 0.4 | 35MFCIVA_1 |
|  |  | 1996 |  | - | 35MF103_1 |
|  |  | 2019 | P162 | −0.5 | 325020190403 |
|  | ESIO | 1995 | P128 | 1.4 | 316N145_5 |
|  |  | 2012 | P153 | 0.4 | 09AR20120105 |
| CDBW^§§^ | WSIO | 1996 | P125 | 0.2 | 320696_3 |
|  |  | 2013 | P125  P162 | 0.2 | 49NZ20130106 |
|  |  | 2019 |  | −0.5 | 49NZ20191229 |
| WSDW^§§§^ | WSIO | 1992 | P114  P127  P134 | 2.0 | 06AQANTX_4 |
|  |  | 1996 |  | 0.8 | 06AQANTXIII_4 |
|  |  | 1999 |  | 0.3 | 06AQ199901_2 |
|  |  | 2010 | P152  P152 | −0.1 | 06AQ20101128 |
|  |  | 2014 |  | −0.1 | 06AQ20141202 |
| ALBW^†^ | ESIO | 1994 | P123  P123  P128  P128 | 0.7 | 09AR9407_1 |
|  |  | 1995 |  | 0.7 | 09AR9404_1 |
|  |  | 1996 |  | 1.4 | 09AR9604_1 |
|  |  | 1996 |  | 1.4 | 09AR9601_1 |
|  |  | 2011 | P149  P161 | 0.7 | 09AR20110104 |
|  |  | 2018 |  | −0.2 | 096U20180111 |
| RSBW^††^ | ESIO | 1992 | P108 | 1.7 | 90KDIOFFE6_1 |
|  |  | 2011 | P152  -  P161 | −0.1 | 320620110219 |
|  |  | 2017 |  | - | 320620170410 |
|  |  | 2018 |  | −0.2 | 320620180309 |

**Supplementary Table 4. CDBW and ALBW properties (θ of CDBW and ALBW, and S_P_ of CDBW) for shift to consider seasonal variation based on previously reported time series data ^[9,11]^.**

| Water of concern | CTD Observation date  (YYYYMM) | (1) θ and S_P_ of source waters observed in cruise season  (θ in ^°^C, S_P_) | (2) θ and S_P_ seasonal climatology of Source waters from mooring in cruise season  (θ in ^°^C, S_P_) | (3) θ and S_P_ of source waters in SON  (θ in ^°^C, S_P_) | (4) θ and S_P_ anomaly of source waters in cruise season  (θ in ^°^C, S_P_);  (1)-(2) | (5) Averaged S_P_ anomaly of source waters  (S_P_) | (6) Corrected θ of source waters through seasonal climatology from mooring in SON (^°^C);  (3)+(4) | (7) Corrected S_P_ of source waters through seasonal climatology from mooring in SON;  (3)+(4)-(5) |
| --- | --- | --- | --- | --- | --- | --- | --- | --- |
| CDBW | 201901 | −0.44,  34.644 | −0.59,  34.638 | −0.69,  34.641 | +0.15,  +0.006 | 0.012 | −0.54 | 34.635 |
|  | 201301 | −0.42,  34.654 | −0.59,  34.638 |  | +0.17,  +0.016 |  | −0.52 | 34.645 |
|  | 199603 | −0.35,  34.659 | −0.47,  34.644 |  | +0.12,  +0.015 |  | −0.57 | 34.644 |
| ALBW | 201802 | −0.58,  - | −0.48 | −0.70 | −0.10 |  | −0.80 |  |
|  | 201101 | −0.57,  − | −0.46 |  | −0.11 |  | −0.81 |  |
|  | 199608 | −0.92,  − | −0.87 |  | −0.05 |  | −0.75 |  |
|  | 199603 | −0.50 | −0.53 |  | +0.03 |  | −0.67 |  |
|  | 199501 | −0.67 | −0.46 |  | −0.21 |  | −0.91 |  |
|  | 199402 | −0.56 | −0.48 |  | −0.08 |  | −0.77 |  |

Abbreviations: ALBW, Adélie Land Bottom Water; CDBW, Cape Darnley Bottom Water
